# Ranking treatments in frequentist network meta-analysis works without resampling methods

**DOI:** 10.1186/s12874-015-0060-8

**Published:** 2015-07-31

**Authors:** Gerta Rücker, Guido Schwarzer

**Affiliations:** Institute for Medical Biometry and Statistics, Medical Center – University of Freiburg, Stefan-Meier-Strasse 26, Freiburg, 79104 Germany

**Keywords:** Network meta-analysis, Ranking, ‘Probability of being best’-statistic, Surface under the cumulative ranking, SUCRA, p-value, AUC

## Abstract

**Background:**

Network meta-analysis is used to compare three or more treatments for the same condition. Within a Bayesian framework, for each treatment the probability of being best, or, more general, the probability that it has a certain rank can be derived from the posterior distributions of all treatments. The treatments can then be ranked by the surface under the cumulative ranking curve (SUCRA). For comparing treatments in a network meta-analysis, we propose a frequentist analogue to SUCRA which we call P-score that works without resampling.

**Methods:**

P-scores are based solely on the point estimates and standard errors of the frequentist network meta-analysis estimates under normality assumption and can easily be calculated as means of one-sided p-values. They measure the mean extent of certainty that a treatment is better than the competing treatments.

**Results:**

Using case studies of network meta-analysis in diabetes and depression, we demonstrate that the numerical values of SUCRA and P-Score are nearly identical.

**Conclusions:**

Ranking treatments in frequentist network meta-analysis works without resampling. Like the SUCRA values, P-scores induce a ranking of all treatments that mostly follows that of the point estimates, but takes precision into account. However, neither SUCRA nor P-score offer a major advantage compared to looking at credible or confidence intervals.

**Electronic supplementary material:**

The online version of this article (doi:10.1186/s12874-015-0060-8) contains supplementary material, which is available to authorized users.

## Background

An increasing number of systematic reviews use network meta-analysis to compare three or more treatments to each other even if they have never been compared directly in a clinical trial [1–4]. The methodology of network meta-analysis has developed quickly and continues to be refined using both Bayesian and frequentist approaches. Bayesian methods are often preferred in network meta-analysis for their greater flexibility and more natural interpretation. It has been argued that ‘Bayesian methods have undergone substantially greater development’ [3, 5]. One outstanding feature of the Bayesian approach often noted is that it allows to rank the treatments according to their comparative effectiveness [6–9]. From a Bayesian perspective, parameters such as those describing the relative effectiveness of two treatments are random variables and as such have a probability distribution. Thus statements such as ‘treatment A is superior to treatment B with probability 60 %’ or ‘Treatment A ranges under the three best of ten treatments with probability 80 %’ are possible. By contrast, from a frequentist perspective, treatment effects are thought as fixed parameters and thus, strictly speaking, a concept like ‘the probability that A is better than B’ does not make sense.

Within the Bayesian framework, authors have noted that it is not sufficient and can be misleading to solely look at the probability of being best, as it does not take uncertainty into account [7–16]. Salanti et al., introducing a rank statistic, extended the consideration to the probabilities that a treatment out of *n* treatments in a network meta-analysis is the best, the second, the third and so on until the least effective treatment [6]. They also introduced several graphical presentations of ranking, such as rankograms, bar graphs and scatterplots [10, 17], and a numerical summary of the rank distribution, called the Surface Under the Cumulative RAnking curve (SUCRA) for each treatment [6, 18, 19]. WinBUGS code for obtaining rank probabilities is given in the supplementary information of [20].

### Objective

In this article, we intend a critical appraisal of ranking, considering both the Bayesian and the frequentist perspective. We use a simple analytical argument to show that the probability of being best can be misleading if we compare only two treatments. For comparing more than two treatments, we explain the SUCRA statistic and introduce a quantity, called P-score, that can be considered as a frequentist analogue to SUCRA. We demonstrate that the numerical values are nearly identical for a data example. Finally we argue that both SUCRA and P-score offer no major advantage compared to looking at credible or confidence intervals.

### Data

Our first real data example is a network of 10 diabetes treatments including placebo with 26 studies, where the outcome was HbA1c (glycated hemoglobin, measured as mean change or mean post treatment value) [21]. These data are provided with R package netmeta [22].

The second real data example is a network of 9 pharmacological treatments of depression in primary care with 59 studies (including 7 three-arm studies), where the outcome was early response, measured as odds ratio (OR) [23].

## Methods

Suppose a network meta-analysis has been conducted using Bayesian methods. We first consider two treatments A and B. Let *μ*_*A*_ and *μ*_*B*_ be independent estimates representing the arm-based effects of treatments *A* and *B*, respectively, as estimated in the network meta-analysis. Let the effects be scaled thus that higher values represent better success. We are interested in the probability that A is more effective than B, that is we want to compute *P*(*μ*_*A*_>*μ*_*B*_).

### Independent normally distributed posteriors

For simplicity, let us assume normal distributions for the posteriors, precisely let $\mu _{A} \sim N(\hat {\mu }_{A}, {\sigma _{A}^{2}}), \mu _{B} \sim N(\hat {\mu }_{B}, {\sigma _{B}^{2}})$. Then the distribution of *μ*_*A*_−*μ*_*B*_ is normal with expectation $\hat {\mu }_{A} - \hat {\mu }_{B}$ and variance ${\sigma _{A}^{2}} + {\sigma _{B}^{2}}$ and we have

$${\fontsize{9.5}{6}\begin{aligned}  P\left(\mu_{A} > \mu_{B}\right) &= P\left(\mu_{A} - \mu_{B} > 0\right)\\ &= 1 - \Phi\left(-\frac{\hat{\mu}_{A} - \hat{\mu}_{B}}{\sqrt{{\sigma_{A}^{2}} + {\sigma_{B}^{2}}}}\right) = \Phi\left(\frac{\hat{\mu}_{A} - \hat{\mu}_{B}}{\sqrt{{\sigma_{A}^{2}} + {\sigma_{B}^{2}}}}\right) \end{aligned}} $$

where *Φ* is the cumulative distribution function (cdf) of the standard normal distribution. It follows that *P*(*μ*_*A*_>*μ*_*B*_)>0.5 is equivalent to $\Phi \left (\left (\hat {\mu }_{A} - \hat {\mu }_{B}\right)/\sqrt {{\sigma _{A}^{2}} + {\sigma _{B}^{2}}}\right) > 0.5$, which is true if and only if $\hat {\mu }_{A} > \hat {\mu }_{B}$, independently of ${\sigma _{A}^{2}} + {\sigma _{B}^{2}}$. In other words, whether A or B is thought more effective (‘better’) depends only on the sign of the difference of the point estimates: the treatment with the greater point estimate wins, regardless of the variances.

#### Fictitious example

**Fig. 1:**
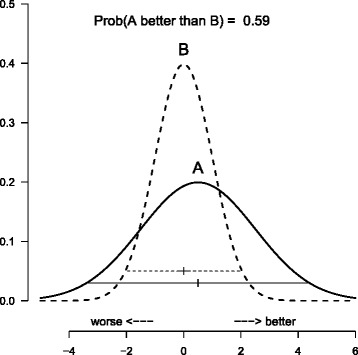
Fictitious example. Two normal posterior distributions following N(0,1) (dashed) and N(0.5, 2^2^) (continuous) with credible intervals. The probability that treatment A, corresponding to the flat distribution, is better than treatment B, corresponding to the steep distribution, is 59 %

Figure 1 shows a fictitious example of two independent normal distributions with means 0.5 and 0 and variances 4 and 1 for treatments A and B, respectively. The theoretical 95 % credible interval of the broader distribution of treatment A (-3.42 to 4.42) completely covers that of the narrower distribution of treatment B (-1.96 to 1.96, dashed). It is natural to conclude that there is no evidence of a difference between the treatments in effectiveness, particularly due to the lack of precision in estimating the effect of A. Note that the densities are cutting each other at two different points: there are regions both to the right and to the left hand side where the density of the flat distribution (treatment A) is greater than that of the distribution of B. In these regions the flat distribution has more mass than the precise distribution, just because it is flat. That is, particularly there is a high probability that A creates unfavorable effects less than −2, that are unlikely to occur under treatment B. Nevertheless, the probability that A is better than B is computed as $\Phi (0.5/\sqrt {5}) = 0.59$. Since this is greater than 0.5, A is thought better than B.

#### ROC curve

The probability *P*(*μ*_*A*_>*μ*_*B*_) can be interpreted as the area under the curve (AUC) for the receiver operating characteristic (ROC) curve defined by

$$R(t) = 1-F_{A}(F_{B}^{-1}(1-t))$$

where *F*_*A*_, *F*_*B*_ are the cdfs of the posterior distributions of *μ*_*A*_ and *μ*_*B*_ (see Additional file 1 for details). In the diagnostic accuracy setting, the AUC provides the probability that, given a randomly selected pair of a diseased and a non-diseased individual, the values of the diseased and the non-diseased individual are in the correct order, e.g., the value of the diseased individual is greater, if higher values indicate illness.

**Fig. 2:**
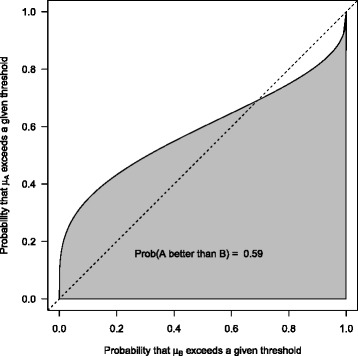
Fictitious example: ROC curve. ROC curve and area under the curve (AUC) corresponding to the example of Fig. 1 (AUC = 0.59)

For Bayesian posterior distributions, the AUC provides the probability that, given that treatment A is truly more effective than treatment B and we randomly select a pair of effect estimates for treatment A and treatment B, A proves better than B. Figure 2 shows the ROC curve and the AUC for the fictitious example. The large difference in variances is reflected by the asymmetric appearance of the curve. Moreover, the curve cuts the dotted line, which is due to the above-mentioned region to the left of Fig. 1 where we observe more unfavorable effects occurring under A. The AUC is 59 %. If this ROC curve would occur from the distribution of a potential diagnostic marker, nobody would trust a diagnostic test based on that marker.

We have seen for normal posterior distributions that the treatment with the more favorable point estimate will be ranked first, regardless of the difference that might be quite small, independently of the variances. If only looking at the ranks, we inevitably ignore the potential difference in precision and length of credible intervals between both posterior distributions.

### Comparing more than two treatments

We now consider a network meta-analysis with *n* treatments and Bayesian posteriors *μ*_*i*_ with means $\hat {\mu }_{i} \ (i=1, \dots, n)$. We cannot assume that the *μ*_*i*_ are independent, as they are all informed by the whole network. We have, however, still an estimate for each difference $\hat {\mu }_{i} - \hat {\mu }_{j}$ with standard deviation *σ*_*ij*_. Again assuming normality for the posteriors, we see as above

(1) $$\begin{array}{@{}rcl@{}}  P(\mu_{i} > \mu_{j}) = \Phi\left(\frac{\hat{\mu}_{i} - \hat{\mu}_{j}}{\sigma_{ij}}\right) \end{array} $$

where *Φ* is the cdf of the standard normal distribution. It follows that the order induced to all treatments by pairwise comparing two treatments preserves the order of the means, independently of the variances. However, the variances enter the above equation and trigger the distance between the underlying probabilities *P*(*μ*_*i*_>*μ*_*j*_): the greater the variances compared to the difference, the more the argument in (1) tends to zero and the more *P*(*μ*_*i*_>*μ*_*j*_) tends to 0.5.

### Surface under the cumulative ranking (SUCRA)

We here recapitulate the definition and interpretation of the SUCRA probabilities introduced by Salanti et al. [6]. First, based on the Bayesian posterior distributions, for each treatment *i*(*i*=1,…,*n*) the probability *P*(*i*,*k*) that treatment *i* has rank *k*(*k*=1,…,*n*) is computed. For each treatment *i*, these rank probabilities form a discrete distribution, as $\sum _{k=1}^{n} P(i,k) = 1$. The cdfs for these distributions can be obtained by

$$F(i,r) = \sum\limits_{k=1}^{r} P(i,k) $$

(*r*=1,…,*n*). *F*(*i*,*r*) gives the probability that treatment *i* has rank *r* or better and we have *F*(*i*,*n*)=1 for all *i*. The surface under the cumulative ranking distribution function for treatment *i* is then defined by

$$\text{SUCRA}(i) = \frac{1}{n-1}\sum\limits_{r=1}^{n-1} F(i,r). $$

To give an interpretation of SUCRA(*i*), we remember that the expectation of a discrete non-negative random variable with values 1,…,*n* can be expressed by the area between the cdf *F* and 1. For the mean rank we have therefore

$$\begin{array}{@{}rcl@{}} \mathrm{E}(\text{rank}(i))& = &n - \sum\limits_{r=1}^{n-1} F(i,r) \\ & = &n - (n-1)\text{SUCRA}(i) \end{array} $$

whence we obtain

$$\text{SUCRA}(i) = \frac{n - \mathrm{E}(\text{rank}(i))}{n-1}. $$

It follows that SUCRA(*i*) is the inversely scaled average rank of treatment *i*, scaled such that it is 1 if E(rank(*i*))=1 (that is, *i* always ranks first) and 0 if E(rank(*i*))=*n* (that is, *i* always ranks last) [6, 19].

SUCRA(*i*) can also be interpreted as the average proportion of treatments worse than *i*.

The mean SUCRA value is 0.5.

### A frequentist version of SUCRA: The P-score

We now look at equation (1) from a frequentist perspective. In the frequentist setting, instead of observing Bayesian posteriors with means and standard deviations, we suppose to have observed effect estimates, again written $\hat {\mu }_{i}$, and standard errors for all pairwise differences $\hat {\mu }_{i} - \hat {\mu }_{j}$, denoted *s*_*ij*_. Again assuming normality, the equation corresponding to (1) is

$$P_{ij} = \Phi\left(\frac{\hat{\mu}_{i} - \hat{\mu}_{j}}{s_{ij}}\right). $$

We give an interpretation for *P*_*ij*_. Apparently, $(\hat {\mu }_{i} - \hat {\mu }_{j})/s_{\textit {ij}}$ is the signed *z*-score of the contrast between treatments *i* and *j*, conditioned on the standard errors. The two-sided p-value of this comparison is given by

$$p_{ij} = 2\left(1 - \Phi\left(\frac{|\hat{\mu}_{i} - \hat{\mu}_{j}|}{s_{ij}}\right)\right). $$

It represents the probability that an absolute difference of the observed size or larger occurs, given the null-hypothesis of no difference is true. Hence we have

$$P_{ij} = \left\{ \begin{array}{cl} p_{ij}/2, & \text{if}\,\hat{\mu}_{i} \le \hat{\mu}_{j} \\ 1 - p_{ij}/2, & \text{if}\, \hat{\mu}_{i} > \hat{\mu}_{j} \end{array} \right. $$

Thus, *P*_*ij*_ is one minus the one-sided p-value of rejecting the null hypothesis *μ*_*i*_≤*μ*_*j*_ in favor of *μ*_*i*_>*μ*_*j*_. *P*_*ij*_ is at least 0.5 if we observe $\hat {\mu }_{i} \ge \hat {\mu }_{j}$, making it likely that *μ*_*i*_>*μ*_*j*_. *P*_*ij*_ is less than 0.5 if we observe $\hat {\mu }_{i} < \hat {\mu }_{j}$, which makes it less likely that *μ*_*i*_>*μ*_*j*_.

We note that, as often, it seems more natural to interpret *P*(*μ*_*i*_>*μ*_*j*_) in the Bayesian setting than to explain the meaning of *P*_*ij*_ in the frequentist context. Nevertheless, they both result in the same decision rule: the greater *P*_*ij*_, the more certain we are that *μ*_*i*_>*μ*_*j*_, and vice versa. Further we note that we do not claim or need independence of the differences $\hat {\mu }_{i} - \hat {\mu }_{j}$.

We may consider the means

$$\bar{P}_{i} = \frac{1}{n-1}\sum\limits_{j, \ j \ne i}^{n} P_{ij}. $$

As *P*_*ij*_ is interpreted as the extent of certainty that *μ*_*i*_>*μ*_*j*_ holds, we may interpret $\bar {P}_{i}$ as the mean extent of certainty that *μ*_*i*_ is greater than any other *μ*_*j*_, averaged over all competing treatments *j* (*j*≠*i*) with equal weights. In other words, $\bar {P}_{i}$ represents the rank of treatment *i* within the given range of treatments, where 1 means theoretically best and 0 means worst. This corresponds to the interpretation of SUCRA(*i*). We will call $\bar P_{i}$ the *P-score* of treatment *i*. P-scores can be seen as the frequentist equivalent of SUCRA values.

From the definition of *P*_*ij*_ it follows that *P*_*ji*_=1−*P*_*ij*_. Thus the sum over all off-diagonal elements of the matrix (*P*_*ij*_) is *n*(*n*−1)/2. For the mean of the $\bar P_{i}$ we obtain

$$\frac{1}{n}{\sum\limits_{i}^{n}} \bar{P}_{i} = \frac{1}{n(n-1)}{\sum\limits_{i}^{n}}\sum\limits_{j, \ j \ne i}^{n} P_{ij} = 0.5 $$

which is the same as the mean of all SUCRA values. In Additional file 2 we give a formal proof that P-scores and SUCRA values are identical if the true probabilities are known.

## Results

We analyzed both data sets with Bayesian as well as frequentist methods. For the Bayesian analysis, we used WinBUGS in combination with R package R2WinBUGS, and for the frequentist analysis we used function netrank of R package netmeta [24]. All analyses were based on the random effects model.

### Diabetes data

**Fig. 3:**
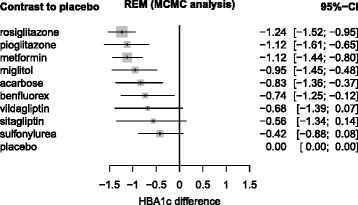
Diabetes data, analyzed with WinBUGS. Diabetes data, analyzed with WinBUGS and ordered by treatment effects (REM = random effects model, MCMC = Markov Chain Monte Carlo analysis with 3 chains, 40000 iterations, 10000 burn in iterations discarded). CI = credible interval (median and 2.5 % / 97.5 % quantiles). The estimated common variance between studies was *σ* ^2^=0.1221

**Fig. 4:**
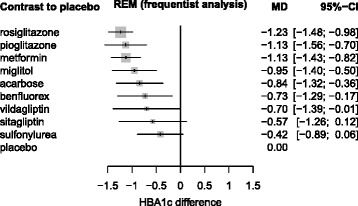
Diabetes data, analyzed with R package netmeta. Diabetes data, analyzed with R package netmeta and ordered by treatment effects (REM = random effects model, CI = confidence interval). The estimated common between study variance was *τ* ^2^=0.1087

First, we report the analysis of the diabetes data given by Senn [21]. The results were similar. Figure 3 shows the results from WinBUGS as a forest plot where all treatments were compared to placebo as a reference, ordered by their medians. Lower values of HbA1c are thought better. Figure 4 shows the corresponding results from netmeta.

The Bayesian rank analysis is based on the probabilities *P*(*i*,*k*) that treatment *i* is the *k*’th best treatment. These are presented in Table 1. Placebo has a probability of 0 to be a good treatment, but a probability of 86 % to be worst. Conversely, rosiglitazone has a probability of (41 + 32 + 17)% = 90 % to be under the best three treatments. Pioglitazone has a higher probability of being best (23 %) compared to metformin (15 %). This is due to its slightly better point estimate, in spite off its clearly lower precision. We have already seen this phenomenon in our fictitious example. However, metformin has the greater probability (15 % + 23 % + 29 % = 67 %) to be under the best three treatments, compared to pioglitazone (23 % + 20 % + 21 % = 64 %).

**Table 1:** Bayesian analysis of the diabetes data [21]. The entry in row *i* and column *k* gives the probability that treatment *i* is the *k*’th best

Rank	1	2	3	4	5	6	7	8	9	10
acar	0.04	0.04	0.06	0.14	0.23	0.25	0.15	0.07	0.02	0.00
benf	0.02	0.04	0.05	0.10	0.16	0.20	0.19	0.15	0.09	0.01
metf	0.15	0.23	0.29	0.20	0.08	0.03	0.01	0.00	0.00	0.00
migl	0.07	0.12	0.13	0.19	0.20	0.14	0.10	0.04	0.01	0.00
piog	0.23	0.20	0.21	0.16	0.09	0.06	0.02	0.01	0.00	0.00
plac	0.00	0.00	0.00	0.00	0.00	0.00	0.00	0.01	0.13	0.86
rosi	0.41	0.32	0.17	0.08	0.02	0.00	0.00	0.00	0.00	0.00
sita	0.03	0.02	0.03	0.04	0.09	0.12	0.17	0.23	0.22	0.05
sulf	0.00	0.00	0.00	0.01	0.02	0.05	0.18	0.30	0.40	0.04
vild	0.05	0.03	0.05	0.08	0.11	0.15	0.18	0.18	0.14	0.04

For the frequentist analysis, Table 2 gives the matrix *P*_*ij*_ of one-sided p-values of rejecting the true null hypothesis of non-inferiority of *i* compared to *j* in favor of the alternative hypothesis that the treatment in the row (*i*) is worse than the treatment in the column (*j*). Small *P*_*ij*_-values mean rejection, that is *i* is worse than *j*. For example, we see that the values in the placebo row all are very small, meaning that it is unlikely that placebo is better than any of the other treatments. Conversely, the values in the rosiglitazone row are all greater than 0.8 except those comparing rosiglitazone with metformin and pioglitazone that are the most promising competitors. When compared to each other, these two are nearly head to head (*P*_*ij*_=0.5), as expected due to their very similar point estimates.

**Table 2:** Frequentist analysis of the diabetes data [21]. The entry in row *i* and column *j* gives one minus the one-sided p-value of rejecting the null hypothesis that the treatment in the row (*i*) is worse than the treatment in the column (*j*) in favor of superiority of *i* compared to *j*

	acar	benf	metf	migl	piog	plac	rosi	sita	sulf	vild
acar	–	0.62	0.13	0.37	0.18	1.00	0.07	0.74	0.95	0.63
benf	0.38	–	0.11	0.28	0.13	0.99	0.05	0.64	0.80	0.53
metf	0.87	0.89	–	0.74	0.50	1.00	0.26	0.93	1.00	0.87
migl	0.63	0.72	0.26	–	0.29	1.00	0.14	0.82	0.94	0.72
piog	0.82	0.87	0.50	0.71	–	1.00	0.32	0.91	0.99	0.85
plac	0.00	0.01	0.00	0.00	0.00	–	0.00	0.05	0.04	0.02
rosi	0.93	0.95	0.74	0.86	0.68	1.00	–	0.96	1.00	0.92
sita	0.26	0.36	0.07	0.18	0.09	0.95	0.04	–	0.64	0.40
sulf	0.05	0.20	0.00	0.06	0.01	0.96	0.00	0.36	–	0.25
vild	0.37	0.47	0.13	0.28	0.15	0.98	0.08	0.60	0.75	–

*Abbreviations*: *acar* acarbose, *benf* benfluorex, *metf* metformin, *migl* miglitol, *piog* pioglitazone, *plac* placebo, *rosi* rosiglitazone, *sita* sitagliptin, *sulf* sulfonylurea alone, *vild* vildagliptin

Table 3 shows the Bayesian and frequentist point estimates (see also Figs. 3 and 4), the SUCRA values and the P-scores (obtained as row means from Table 2), the treatments now ordered with decreasing rank. The results confirm that the ranking mainly depends on the point estimates, with the exception of metformin and pioglitazone that change places, now accounting for the greater precision of metformin. Moreover, we see that SUCRA values and P-scores, in addition to their corresponding interpretation, also have very similar numeric values. R code for the diabetes example is provided in function netrank of the netmeta package, Version 0.8-0 [22].

**Table 3:** Bayesian and frequentist point estimates, SUCRA values and P-scores for the diabetes data [21]

	Point estimates		Ranks	
	Bayesian	Frequentist	SUCRA	P-score
	WinBUGS	netmeta	WinBUGS	netmeta
rosiglitazone	-1.24	-1.23	0.890	0.893
metformin	-1.12	-1.13	0.780	0.782
pioglitazone	-1.12	-1.13	0.773	0.775
miglitol	-0.95	-0.95	0.620	0.614
acarbose	-0.83	-0.84	0.520	0.520
benfluorex	-0.74	-0.73	0.439	0.436
vildagliptin	-0.68	-0.70	0.413	0.423
sitagliptin	-0.56	-0.57	0.334	0.333
sulfonylurea	-0.42	-0.42	0.213	0.210
placebo	0	0	0.018	0.014

### Depression data

**Fig. 5:**
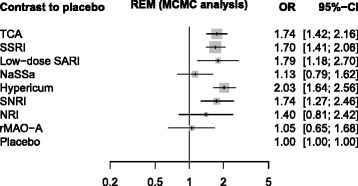
Depression data, analyzed with WinBUGS. Depression data, analyzed with WinBUGS (REM = random effects model, MCMC = Markov Chain Monte Carlo analysis with 3 chains, 40000 iterations, 10000 burn in iterations discarded). CI = credible interval (median and 2.5 % / 97.5 % quantiles). The estimated common between study variance was *σ* ^2^=0.2011

**Fig. 6:**
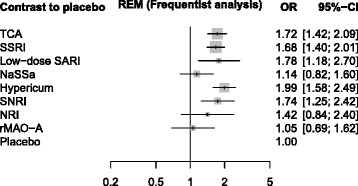
Depression data, analyzed with R package netmeta. Depression data, analyzed with R package netmeta (REM = random effects model, CI = confidence interval). The estimated common between study variance was *τ* ^2^=0.1875

For the depression data [23], the Bayesian MCMC approach (Fig. 5) and the frequentist approach (Fig. 6) showed results slightly more different. Particularly, the point estimates of TCA and SNRI are similar for the Bayesian approach, but different when using our frequentist approach. Accordingly, the ranking differs (Table 4): For the Bayesian approach with SUCRA, TCA benefits from its higher precision, for the frequentist approach (P-score), SNRI benefits from its larger point estimate. We attribute this difference to difference in point estimation rather than the different ranking methods.

**Table 4:** Columns 2-4: Bayesian and frequentist point estimates (OR). Columns 5-7: SUCRA values based on MCMC analysis using WinBUGS (SUCRA-1), P-scores, SUCRA values based on resampling using Stata function mvmeta (SUCRA-2) for the depression data [23]

	Point estimates			Ranks		
	Bayesian	Frequentist		SUCRA-1	P-score	SUCRA-2
	WinBUGS	netmeta	mvmeta	WinBUGS	netmeta	mvmeta
Hypericum	2.03	1.99	1.99	0.897	0.894	0.895
Low-dose SARI	1.79	1.78	1.78	0.714	0.720	0.719
TCA	1.74	1.72	1.72	0.690	0.680	0.680
SNRI	1.74	1.74	1.74	0.681	0.689	0.689
SSRI	1.70	1.68	1.68	0.610	0.616	0.617
NRI	1.40	1.42	1.42	0.447	0.445	0.444
NaSSa	1.13	1.14	1.14	0.207	0.213	0.213
rMAO-A	1.05	1.05	1.05	0.157	0.152	0.152
Placebo	1	1	1	0.096	0.091	0.092

*Abbreviations*: *SARI* serotonin antagonist and reuptake inhibitor, *TCA* tricyclic and tetracyclic antidepressant, *SNRI* serotonin-noradrenaline reuptake inhibitor, *SSRI* selective serotonin reuptake inhibitor, *NRI* noradrenaline reuptake inhibitor, *NaSSa* specific serotonergic antidepressant agents, *rMAO-A* reversible inhibitors of monoaminoxidase A

We analysed these data with a third approach, the frequentist resampling method by White et al. [25, 26]. In the mvmeta function of Stata, rankings are constructed via a parametric bootstrap procedure in analogy to drawing from a Bayesian posterior distribution. For each parameter vector drawn from the multivariate distribution, the treatment that ranks first is identified, and the probability of being best for each treatment is estimated by the proportion of samples where this treatment ranks first. SUCRA values are calculated as for the Bayesian approach. The results for the depression data were very similar to those of our own method. The point estimates were identical and the SUCRA values nearly identical to the P-Score values. This corroborates our conclusion that both P-scores and SUCRA values are mainly driven by the point estimates and that P-scores are a good approximation to values generated by resampling methods.

## Discussion

It has been argued that ranking treatments by the probability of being best and SUCRA is an originally Bayesian concept, and this has been claimed to be a reason to prefer Bayesian methodology when performing network meta-analysis [3, 7, 9]. In this article, we reassessed these arguments. First, we have shown that for the normal distribution the probability *P*(*μ*_*A*_>*μ*_*B*_) is larger than *P*(*μ*_*B*_<*μ*_*A*_) if and only if the expectation of *μ*_*A*_ is greater than that of *μ*_*B*_. Though the probabilities depend on the variances, the ranking order does not. We gave a fictitious example where there was no evidence of a relevant difference between treatments A and B. The correct interpretation is that the uncertainty in estimating the effect of A is too large to make us take the slightly better point estimate very serious, and we should attribute this slight superiority to the lack of precision. We compared the situation to the diagnostic test setting, where the AUC measures the probability that two values of a marker are in correct order. It is known that ROC curves may become asymmetrical with respect to the diagonal if one distribution has a much greater variance than the other. In extreme cases of one distribution with long tails in either direction, the AUC makes no sense anymore.

Further, we introduced a frequentist analogue to SUCRA. It is based solely on the point estimates and standard errors of the frequentist network meta-analysis estimates. From these, we derived P-scores that represent means of one-sided p-values under normality assumption. The P-scores have an interpretation analogous to the SUCRA values and measure the extent of certainty that a treatment is better than another treatment, averaged over all competing treatments. The numerical values of SUCRA and P-score were similar. Like the SUCRA values, the P-scores induce a ranking of all treatments that mostly follows that of the point estimates, but takes precision into account.

It is important to consider the numerical values themselves, not only their ranks. For both our examples, there are treatments (rosiglitazone and hypericum, respectively) with an average probability of 89 % of being superior to a competing treatment. These values are considerably high, but they do not exceed 90 % or 95 %. Also in both examples, some other treatments have ranks quite similar to each other. We have shown that the mean value of the P-scores is always 0.5; however, the variance may vary greatly. All P-score values may just as well scatter tightly around 50 %, indicating that all treatments are of similar efficacy. This is the case for the example of dietary fat given in the supplement of [20], where the P-scores for three treatments are 0.58 (diet 2), 0.51 (diet 1) and 0.41 (control). In such a case, simple ranks are likely to be misinterpreted.

Salanti [1] criticized that ‘Presentation of results on the basis of the statistical significance of pairwise comparisons, as suggested by Fadda et al. [27], may be misleading as it overemphasizes the importance of p-values’. We have shown that, somewhat ironically, a concept like SUCRA that originates from a Bayesian point of view has a frequentist analogue that in fact is simply based on p-values.

P-values are frequently used in a different context when ranking very large gene lists in gene expression analysis and genome-wide association studies where very small two-sided p-values indicate different gene expression between groups of patients [28–30]. By contrast, our approach leads to sums of one-sided p-values where large values indicate higher-ranking treatments.

Kibret et al. in a simulation study [9] have shown that unequal numbers of studies per comparison resulted in biased estimates of treatment rank probabilities. The expected rank was overestimated for treatments that were rarely investigated and underestimated for treatments occurring in many studies. This finding is probably due to the differences in precision of estimates between rare and frequent treatments.

Jansen et al. [31] mentioned the possibility to ‘approximate the results of a Bayesian analysis […] in a frequentist setting’, but did not descibe details. One possible choice is the mvmeta function of Stata we applied to our second example.

With this method, a data augmentation step was necessary to impute data for a chosen reference treatment for all studies even if they did not have that treatment arm [25].

To the best of our knowledge, a simple analytical method like ours, based on frequentist p-values and bypassing the probabilities of being k’th best, has not been described.

In this article, we limited our considerations to the normality assumption, because in frequentist statistics confidence intervals usually are based on a normal or t-distribution assumption. In the Bayesian framework, posterior distributions, though depending on prior assumptions, are not restricted to be normal, particularly, they may be skew. We did not investigate the behaviour of the ranking probabilities for skewed or other types of distributions.

In a Bayesian context, probably the most straightforward question with respect to ranking treatments is the probability of each treatment being best. However, the concept is not so straightforward from the frequentist perspective. We explicitly note that here lies a difference between our approach and others: we completely avoid to compute ranking probabilities (i.e., the probability of being best, second-best, and so on). Because of the dependence between all NMA estimates, this would be difficult or even impossible without resampling methods. We replace this by looking at all pairwise comparisons. These are easy to implement, because independence is not needed in the first step when computing the p-values of the contrasts. We do not sum up independent quantities when summing up the p-values in the second step, as they all rely on estimation of the network as a whole. Nevertheless, it turns out that the interpretation of this sum is quite similar to the interpretation of SUCRA: for treatment *i*, it is the mean certainty that treatment *i* is better than another treatment *j*. In a way, looking at all pairwise comparisons is a trick for getting a ranking list without asking for the probability of being k’th under n.

Ranking, however done, depends on the criteria. In both our examples, this was the primary efficacy outcome of the NMA. In practice there are almost always multiple outcomes. A treatment may be best for efficacy, but worst for safety, or best for short-term survival, but worse for long-term survival.

Before ranking treatments, we have to choose criteria, or we may give separate ranking lists for different outcomes, or we may combine several criteria to a joint score. The problem is known from diagnostic testing, where a trade-off between sensitivity and specificity is made, e.g., by taking their sum (equivalent to the Youden index) or a weighted sum with a combination of prevalence and utilities as weights.

We distinguish two issues: the choice of the outcome and how to rank treatments, given the outcome is fixed. In the present paper, we only looked at the second topic, assuming that a specific outcome has been selected beforehand.

## Conclusions

We introduced a frequentist analogue, called P-scores, to the SUCRA concept in Bayesian network meta-analysis methodology. Whereas Bayesian ranking is based on the posterior distribution, P-scores are based on the frequentist point estimates and their standard errors. Both concepts, the Bayesian SUCRA and the frequentist P-scores, allow ranking the treatments on a continuous 0-1 scale. The numerical values are similar. We should keep in mind that, at least under normality assumption, the order depends largely on the point estimates. Simply ranking treatments based on SUCRA or P-scores has no major advantage compared to ranking treatments by their point estimates. The values themselves of the P-score should be taken into account. Precision should also be taken into account by looking at credible intervals or confidence intervals, whether one opts for ranking or not. When reporting a network meta-analysis, we recommend that authors should always present credible or confidence intervals, for example in form of a forest plot comparing all treatments to a chosen reference.

## Availability of supporting data

The diabetes data are published [21] and also available as part of R package netmeta [22]. The depression data are published in the supplemental material of [23], Fig. 1. The dietary fat data are provided in the supplement of [20]. Ethical approval was not necessary, as this is a methodological study.

## Additional files

Additional file 1
**Probability of being best and AUC.** Contains details of the relation between the probability of being best and the area under the curve (AUC). (PDF 92 kb)

Additional file 2
**Proof that SUCRA and P-score have identical values.** Contains a formal proof that the values of SUCRA and P-score are identical if the true probabilities are known. (PDF 88 kb)

## Abbreviations

AUC: Area under the curve
CI: Confidence interval/credible interval
cdf: Cumulative distribution function
HbA1c: Glycated hemoglobin
MCMC: Markov Chain Monte Carlo
NMA: Network meta-analysis
OR: Odds ratio
REM: Random effects model
ROC: Receiver operating characteristic
SUCRA: Surface under the cumulative ranking Drug names are explained at the places where they occur in the text
