# Supplementary material for: Ranking treatments in frequentist network meta-analysis works without resampling methods
Source: BMC Med Res Methodol. 2015 Jul 31;15:58. doi: 10.1186/s12874-015-0060-8 (PMC4521472; doi:10.1186/s12874-015-0060-8)
Supplement: Additional file 1 — Probability of being best and AUC. Contains details of the relation between the probability of being best and the area under the curve (AUC). (PDF 92 kb) [file 12874_2015_60_MOESM1_ESM.pdf]

Additional file to:
Ranking treatments in frequentist network meta-analysis works without re-sampling methods

Gerta Rücker and Guido Schwarzer

Probability of being best and AUC

We have

$$P(\mu_A > \mu_B) = \int_{-\infty}^{\infty} P(\mu_A > y)\; dP_{\mu_B} = \int_{-\infty}^{\infty} (1 - F_A(y))\; dP_{\mu_B}.$$

Writing f_B for the density of μ_B, we obtain

$$P(\mu_A > \mu_B) = \int_{-\infty}^{\infty} (1 - F_A(y)) f_B(y)\; dy.$$

Substituting y by t with $t = 1 - F_B(y)$, we obtain $y = F_B^{-1}(1-t)$ and $dt = -f_B(y)\; dy$ and thus

$$P(\mu_A > \mu_B) = \int_0^1 1 - F_A(F_B^{-1}(1-t))\; dt.$$

Interpreting

$$R(t) = 1 - F_A(F_B^{-1}(1-t))$$

as a receiver operating characteristic (ROC) function characterizing the distance of the distributions F_A and F_B, we see that

$$P(\mu_A > \mu_B) = \int_0^1 R(t) dt$$

corresponds to the area under the ROC curve (AUC).
