# Supplementary material for: Ranking treatments in frequentist network meta-analysis works without resampling methods
Source: BMC Med Res Methodol. 2015 Jul 31;15:58. doi: 10.1186/s12874-015-0060-8 (PMC4521472; doi:10.1186/s12874-015-0060-8)
Supplement: Additional file 2 — Proof that SUCRA and P-score have identical values. Contains a formal proof that the values of SUCRA and P-score are identical if the true probabilities are known. (PDF 88 kb) [file 12874_2015_60_MOESM2_ESM.pdf]

Additional file to:
Ranking treatments in frequentist network meta-analysis works without resampling methods

Gerta Rücker and Guido Schwarzer

Proof that SUCRA and P-score are identical

We consider all probabilities as known. Let $\mathsf{R}(i) = k$ mean s that treatment i has rank k. We have

$$P_{ij} = \sum_{k=1}^{n-1} \sum_{l=k+1}^{n} P(\mathsf{R}(i) = k \wedge \mathsf{R}(j) = l)$$

and

$$(n-1)SUCRA(i) = \sum_{r=1}^{n-1} F(i,r) = \sum_{r=1}^{n-1}\sum_{k=1}^{r} P(i,k) = \sum_{k=1}^{n-1}\sum_{r=k}^{n-1} P(i,k) = \sum_{k=1}^{n-1}(n-k)P(i,k)$$

which is the expected proportion of treatments worse than i. It follows

$$\begin{aligned}\sum_{j=1}^{n} P_{ij} &= \sum_{j=1}^{n}\sum_{k=1}^{n-1}\sum_{l=k+1}^{n} P(\mathsf{R}(i) = k \wedge \mathsf{R}(j) = l) \\ &= \sum_{k=1}^{n-1}\sum_{l=k+1}^{n} P(i,k) = \sum_{k=1}^{n-1}(n-k)P(i,k) \\ &= (n-1)SUCRA(i)\end{aligned}$$

and thus

$$\bar{P}_i = \frac{1}{n-1}\sum_{j=1}^{n} P_{ij} = SUCRA(i)$$

which is what we wanted to prove.

Note: For $n > 2$, neither the ranking probabilities $P(i,k)$, nor the probabilities P_{ij} can be uniquely derived from $\bar{P}_i$ or $SUCRA(i)$.
